# Plant and Animal Protein Intakes Largely Explain the Nutritional Quality and Health Value of Diets Higher in Plants: A Path Analysis in French Adults

**DOI:** 10.3389/fnut.2022.924526

**Published:** 2022-06-28

**Authors:** Elie Perraud, Juhui Wang, Marion Salomé, Jean-François Huneau, Nathanaël Lapidus, François Mariotti

**Affiliations:** ^1^Université Paris-Saclay, AgroParisTech, INRAE, UMR PNCA, Paris, France; ^2^Sorbonne Université, INSERM, Institut Pierre Louis d'Epidémiologie et de Santé Publique IPLESP, AP-HP Sorbonne Université, Public Health Department, Saint-Antoine Hospital, Paris, France

**Keywords:** plant-based diet, path analyses, protein, diet quality, nutrient

## Abstract

Diets higher in plants are associated with lower risks of chronic diseases. However, animal foods, which are rich in protein, are also rich in some important minerals and vitamins. Using data from a representative survey in France (INCA3, *n* = 1,125), we used path analyses as a mediation-like approach to decipher the importance of plant and animal proteins in the relationship between the plant-based diet index (PDI) and diet quality. We used three types of diet quality scores, namely, nutrient security, positive nutrient adequacy, and long-term mortality risk of four diet-related diseases (i.e., coronary heart disease, stroke, type 2 diabetes, and colorectal cancer). We identified positive and negative mediations, i.e., changes in plant/animal protein intake that are associated with changes in PDI, and favor or limit the association with the diet quality score. The PDI was positively associated with the risk of long-term mortality but not significantly with nutrient adequacy or nutrient security. A positive mediation by plant protein was found for all diet quality scores (specific indirect effects (SIEs) ranging from 0.04 to 0.10 SD). Conversely, the association between PDI and nutrient adequacy (but not nutrient security) was negatively mediated by animal protein intake (SIE: −0.06 SD). In further detailed models, the association between PDI and diet quality was mainly positively mediated by protein foods from the fruit-vegetables-legumes group (0.01 SD for the nutrient security and 0.02 SD for the nutrient adequacy) and whole grains (0.02 SD for the nutrient adequacy). Our data suggest that the positive impact of plant-based diets on diet quality is largely driven by higher intakes of plant protein foods, especially from fruits-vegetables-legumes and whole grains. Conversely, lower animal protein intake tends to limit the positive impact of plant-based diets on overall positive nutrient adequacy but not security. Protein sources appear critical to healthy plant-based diets.

## Introduction

In Western countries, there is an ongoing trend toward plant-based diets, which tend to favor plant protein for replacing animal sources for sustainability reasons, including health and environmental impacts (1, 2). Similarly, most food-based dietary guidelines worldwide advise increasing the consumption of food groups based on plant sources and decreasing those based on animal sources (3, 4). This mainly consists in recommending a greater consumption of fruits, vegetables, legumes, and nuts and lower consumption of meat, particularly red and processed meat (3). These recommendations originate from a corpus of evidence that combines dietary and nutrient levels. For example, the importance of iron (5) or saturated fatty acid (6, 7) in the risk of chronic disease has been much studied. At the overall pattern level or food group level, there is also evidence regarding the risks of developing non-communicable diseases. For instance, a high intake of red meat is considered to increase the risk of colorectal cancer, cardiovascular diseases, and type 2 diabetes (8–10). Studies also argue the importance of some food groups for securing sufficient nutrient intake and avoiding deficiency (11, 12).

Some historically plant-based diets, such as vegetarian diets, have been associated with healthier food choices (13), leading to lower risks of cardiovascular disease (14), cerebrovascular disease, type 2 diabetes, and chronic kidney disease (15). Although risks of deficiency for protein and amino acids are small to inexistent in vegetarians, some studies have shown some risk of insufficient intake and deficiency for some vitamins and minerals (e.g., vitamin B12), depending on the type of plant-based diet (15–17).

Because plant-based diets broadly vary in nutritional quality, Satija et al. (18) developed the plant-based diet index (PDI), which combines the consumption of 18 dietary groups to quantify adherence to plant-based diets. Accordingly, there are two versions of the PDI, namely, the healthful and the unhealthful PDI, based on the alleged healthiness of the plant/animal food groups (19).

The protein component of a diet, whether from plant or animal foods, could be central to the nutritional quality of plant-based diets because protein food groups are associated with nutrient adequacy and long-term health (20, 21). Proteins are a big part of dietary guidelines, and they reflect the current shift in the Western diet toward one that is more plant-based. However, it remains unknown how diet quality is affected by a higher adherence to a plant-based diet according to the background change in plant/animal protein intake. In structural equation modeling, path analysis is often used to analyze supposedly causal relationships for identifying mediators. Yet, path analysis does not require a causal relationship and it can be used to account for multicollinearity and identify the importance of some factors in a relationship between two variables (22). Here, we used path analysis to explore how the animal/plant origin of protein and other diet components could explain the relationship between PDI and diet quality, considered as both nutrient adequacy/security and long-term health.

## Subjects and Methods

### Study Population

Data were used from the third individual-based national study on food consumption survey (INCA3) performed in France between 2014 and 2015 (23, 24). This survey is a representative of the French population. Women between 18 and 54 years and men between 18 and 64 years were included in the study. Older adults were excluded because some of their nutrient requirements differ from those of younger adults and, in particular, after menopause for women (25, 26). Participants identified as being under-reporters were excluded by first using the basal metabolic rate as estimated by the Henry equation (27) and then applying the cutoff values recommended by Black (28). The final sample contained 1,125 adults (Supplemental Figure 1).

Data were collected by three non-consecutive 24-h dietary recalls (two during the week and one at the weekend) over a 3-week period. The nutrient contents of the foods were extracted from the 2016 database of the *French Center information sur la Qualité des Aliments* (CIQUAL) (29). Using recipes, foods were reclassified into different food groups for further analyses.

### Protein

Each food was classified as either plant, animal, or mixed protein sources. Using the percentage of animal and plant protein calculated for mixed dishes using recipes, we assessed individual plant and animal protein intakes.

Using protein foods, protein source groups were then created. As previously defined (30), protein foods were considered foods containing both >10% energy as protein and conveying >5 g protein in the diet of high consumers (90th percentile). Protein foods were then classified into eleven groups, namely, refined grains, whole grains, dairy, eggs, beef/sheep meat, poultry, pork, processed meat, fish, fruits-vegetables-legumes, and others. These groups were defined as conventional nutritional groups (e.g., refined grains and whole grains) and as a grouping of a sufficient number of protein foods consumed by the participants. Nuts were not included as a distinct group due to the very low consumption of this food group in the study population. Note that the latter explains grouping protein food from fruit, vegetables, and legumes. The protein intake from each of these groups was then calculated.

### Level of Adherence to a Plant-Based Diet

We used the PDI as developed by Satija et al. (18) to assess the level of adherence to an overall plant-based diet. This score was calculated using the number of servings for 18 food groups (18, 19). These 18 food groups were created based on nutrient and culinary similarities. Animal food groups were given negative scores and plant food groups were given positive scores. Each of the 18 food groups was divided into quintiles of consumption. A score between 1 and 5 was assigned to each quintile. For each plant food, participants received a score of 1 for consumption below the lowest quintile; a score of 2 if the plant food consumption was between the lowest and the second-lowest quintile; and so on. If the consumption of the plant food was above the highest quintile, a score of 5 was assigned to the participant. In addition, participants receive a score of 5 for consumption of animal protein below the lowest quintile; a score of 4 if the animal food consumption was between the lowest and the second-lowest quintile; and so on.

### Diet Quality Scores

Three diet quality scores were used to assess the different effects on health. The higher these scores are, the better the quality. We used the SecDiet to evaluate nutrient security. This score is based on the consumption of twelve critical nutrients (31). For each nutrient, we calculated the probability of having sufficient-enough intake to avoid overt nutrient deficiency, using the probability distribution of the standard normal distribution of nutrient requirements while taking into account the mean intake, the day-to-day intake variability, the inter-individual variability, and the nutrient reference value (31). Supplemental Method 1 describes more precisely the calculation of the SecDiet.

We used the adequacy subscore of the PANDiet (AS) to evaluate the nutrient adequacy. PANDiet is a 100-point probabilistic score evaluating adequate overall nutrient intake. The adequacy subscore, used in this study, is calculated as the average probability of adequacy for 28 micro and macronutrients whose usual intake should be above its reference value (30, 32). Supplemental Method 2 describes more precisely the calculation of the AS of the PANDiet.

The HiDiet was used to assess the diet impact on long-term mortality and morbidity. The HiDiet score is based on the principle of the comparative risk assessment but applied to the risk of an individual. It is an individual version of the Evaluate the Potential Impact of a Diet (EpiDiet) (33, 34). The conceptual basis and methodological foundation of the two models are the same. They are laid out in the comparative risk assessment framework, allowing for evaluating the potential impacts of dietary changes on the long-term morbidity and mortality caused by some diet-related diseases.

In this study, we set up the HiDiet model with values reported in a series of validated international meta-analyses published by a European team (35–39). As previously described (33), we selected 12 diet-related factors (i.e., consumption of fruits, vegetables, nuts or seeds, whole grains, unprocessed red meats, processed meats, sugar-sweetened beverages, fish, dairy products, eggs, refined grains, and legumes) and four diet-related diseases (i.e., coronary heart disease, stroke, type 2 diabetes, and colorectal cancer).

The reference population used was the French population in 2014, stratified into subpopulations by 5-year age groups and sex. The population demographics and national disease-specific deaths were provided by the National Institute of Statistics and Economic Studies (40) and the Epidemiological Center on Medical Causes of Death (41), respectively. For each individual, two dietary scenarios were formed. The baseline scenario corresponds to the average daily intake of each food and beverage group consumed per capita in the subpopulation, and the counterfactual one to the distribution of the daily amount of each food and beverage group consumed by the individual during the survey.

### Statistical Analyses

Path analyses were used to assess how protein sources could mediate the effect of PDI on diet quality scores. Note that the word “effect” is standard terminology for path analysis, but it refers to association, not causation (42). Path analysis relies on a hypothetical structural representation of relationships between dependent or independent variables. Graphically, an arrow from variable A to variable B represents the direct effect (DE) of A on B, which can be estimated as a linear regression coefficient. Using an additional set of mediators Z_i_ and accounting for the effects of A to Z_i_ and of Z_i_ to B, it is possible to estimate the direct, indirect (mediated through Z_i_), and total effects of A to B (22, 43) (Supplemental Figure 2). A correlation analysis is performed on the mediators to exclude collinear ones (Supplemental Tables 1, 2).

**Figure 1:**
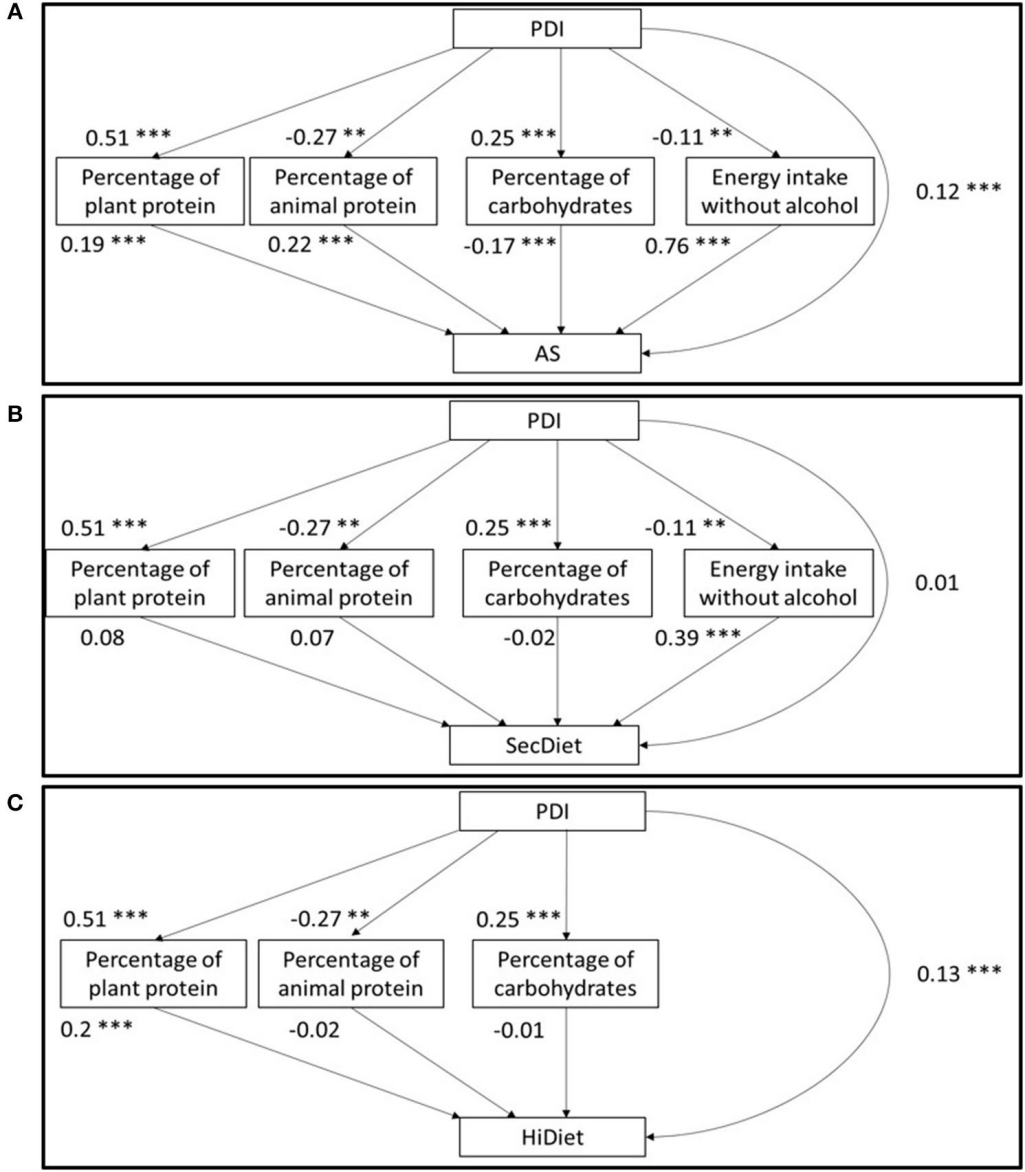
Structural model of the path analyses between the PDI and the AS **(A)**, SecDiet **(B)**, and the HiDiet **(C)** with the percentage of energy from plant and animal proteins, the percentage of energy from carbohydrates, and the energy intake without alcohol as mediators. All regression coefficients are standardized. *P < 0.05, **P < 0.01, ***P < 0.001.

For each diet quality score, two-type models were studied. The first type of models included the energy intake (EI) and the percentage of EI as total carbohydrates without fiber (referred to as “carbohydrates” in the following), animal protein, and plant protein as mediators. These models are named A1 when the diet quality score is the AS, S1 when it is the SecDiet, and H1 when it is the HiDiet. In these models, the PDI is an exogenous variable (not explained by other variables), and the mediators and the diet quality scores are endogenous variables (explained by other variables) (Figure 1). Physical activity was not assessed in this study, so it could not be included in the HiDiet calculation. It was not possible to include EI without physical activity in the calculation because energy balance is the most important factor in the estimation of morbidity and mortality. Thus, EI could not be included in the HiDiet model (H1).

**Figure 2:**
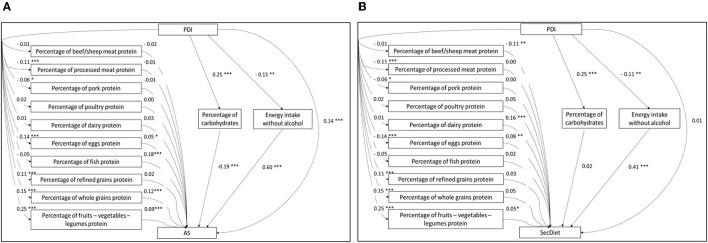
Structural model of the path analyses between the PDI and the AS **(A)** and the path analyses between the PDI and the SecDiet **(B)** with the percentage of energy from whole grains protein (%EI), refined grains protein (%EI), dairy protein (%EI), eggs protein (%EI), beef/sheep meat protein (%EI), pork protein (%EI), poultry protein (%EI), processed meat protein (%EI), fish protein (%EI), fruits-vegetables-legumes protein (%EI), carbohydrates (%EI), and energy intake without alcohol as mediators. All regression coefficients are standardized. *P < 0.05, **P < 0.01, ***P < 0.001.

The second type of models was developed to describe in detail the varied effects of plant and animal protein inside each group. In this model, the percentages of EI from both plant and animal proteins were replaced by the percentage of EI (%E) coming from the protein food groups (Figure 2). However, this particular model was not used with the HiDiet because of the redundancy between the protein food groups in the model and the food groups considered in the HiDiet risk model. Similarly, as the first type of models, A2 is the model with the AS as diet quality score and S2 is the model with the SecDiet as diet quality score.

Standardized regression coefficients were estimated with their standard errors by bootstrap resampling using the lavaan package (44). A coefficient of β for a pathway A to B means that a change of 1 standard deviation (SD) in A translates into a change of β SD in B. Using standardized coefficients, this allows for a direct comparison of the relative magnitude of the estimated effects. Analyses were conducted using the R statistical software version 4.0.3 (45). All tests were two-tailed with a 0.05 significance threshold.

## Results

The descriptive characteristics of the sample (*n* = 1,125) are presented in Supplemental Table 3. Table 1 describes the protein intake by PDI quartiles. Protein intakes of refined grains were similar across quartiles. In the fourth quartile, protein intake from whole grains, fruits, vegetables, and legumes combined was ~80% higher than in the first quartile (7.74 vs. 4.34 g/day). Notably, the first quartile had higher energy, protein, and lipid intakes (Table 1).

**Table 1:** Description (mean ± standard deviation) by quartiles of plant-based diet index of the protein, protein categories, carbohydrates, lipid, and energy without alcohol intake and SecDiet, AS, and HiDiet.

	**Whole sample**	**PDI quartile**				***P* trend**
		**1st**	**2nd**	**3rd**	**4th**	
PDI	35.1 ± 5.39	29.09 ± 3.04	34.7 ± 1.20	38.04 ± 0.88	42.03 ± 2.21	<0.01
Protein (g/day)	90.63 ± 33.24	101.21 ± 41.54	86.42 ± 29.47	89.68 ± 29.94	82.47 ± 24.64	<0.01
**Plant protein**	32.95 ± 12.57	32.56 ± 13.74	31.02 ± 12.23	34.42 ± 12.00	35.31 ± 11.51	<0.01
Refined grains protein	18.54 ± 10.37	19.06 ± 12.34	17.96 ± 10.11	19.06 ± 9.58	18.2 ± 8.65	0.37
Whole grains protein	1.34 ± 3.24	0.91 ± 2.33	1.17 ± 3.10	1.48 ± 3.34	2.13 ± 4.07	<0.01
Fruits-vegetables-legumes protein	4.14 ± 3.46	3.43 ± 3.40	3.4 ± 2.86	4.99 ± 3.96	5.61 ± 3.13	<0.01
**Animal protein**	57.68 ± 26.8	68.64 ± 33.37	55.4 ± 22.83	55.26 ± 23.58	47.16 ± 18.77	<0.01
Beef/sheep meat protein	10.39 ± 14.57	14.28 ± 20.59	9.02 ± 12.88	9.05 ± 10.57	8.07 ± 8.77	<0.01
Processed meat protein	7.66 ± 8.62	9.61 ± 11.01	7.19 ± 8.13	7.88 ± 7.68	5.26 ± 5.61	<0.01
Pork protein	5.46 ± 11.06	6.74 ± 12.80	5.67 ± 13.01	4.55 ± 8.51	4.05 ± 7.00	0.01
Poultry protein	*8.21*±*11.08*	*7.7*±*10.8*	*8.63*±*12.25*	*8.91*±*11.35*	*7.6*±*9.37*	0.36
Dairy products protein	16.76 ± 10.22	19.41 ± 12.04	15.74 ± 9.45	15.68 ± 9.23	15.52 ± 8.90	<0.01
Eggs protein	1.87 ± 3.27	2.42 ± 3.50	1.83 ± 3.57	1.56 ± 3.01	1.41 ± 2.59	<0.01
Fish protein	6.59 ± 9.85	7.67 ± 10.03	6.68 ± 10.06	6.78 ± 10.6	4.61 ± 8.31	<0.01
Energy intake without alcohol (kcal/day)	*2, 272.72*±*741.26*	*2, 454.68*±*863.83*	*2, 159.25*±*708.67*	*2, 247.04*±*688.85*	*2, 211.37*±*610.12*	<0.01
Carbohydrate (g/day)	272.28 ± 101.20	284.18 ± 117.29	258.82 ± 97.26	276.9 ± 97.06	272.13 ± 85.76	<0.01
Lipid (g/day)	85.38 ± 30.58	96.04 ± 35.88	81.08 ± 28.79	80.51 ± 26.70	81.21 ± 24.82	<0.01
SecDiet	0.952 ± 0.081	0.957 ± 0.072	0.944 ± 0.094	0.955 ± 0.079	0.957 ± 0.071	0.06
AS	67.32 ± 13.57	68.56 ± 13.28	65.12 ± 13.8	67.66 ± 13.83	68.81 ± 12.94	<0.01
HiDiet	*0.004*±*0.129*	−*0.020*±*0.121*	*0.000*±*0.134*	*0.010*±*0.133*	*0.039*±*0.119*	<0.01

*PDI, plant diet index; AS, adequacy subscore of the PANDiet*.

In the first three models (A1, S1, and H1), which included the percentage of animal and plant proteins, the total effect between the PDI and the diet quality index was only significant for the HiDiet model. For each 1 SD increase in the PDI, the HiDiet increased by 0.23 SD (Table 2). The association between the PDI and mediators was similar in all models (Figure 1). An increase in 1 SD of the PDI was associated with a 0.51 SD increase in the percentage of plant protein (*P* < 0.001) and a 0.25 SD increase in the percentage of EI from carbohydrates (*P* < 0.001) (Figure 1). Conversely, it was associated with a 0.27 SD decrease in the percentage of animal protein (*P* < 0.001) and a 0.11 SD decrease in the EI (*P* = 0.001) (Figure 1).

**Table 2:** Variables of the path analyses between the PDI and the three diet quality scores (AS, SecDiet, and HiDiet) with the plant protein (percentage of total energy intake, %E), animal protein (%E), carbohydrates (%E), and energy intake without alcohol as mediators.

**Effects**	**AS model (A1)**			**SecDiet model (S1)**			**HiDiet model (H1)**		
	**Standardized coefficient**	**SE**	***P*-Value**	**Standardized coefficient**	**SE**	***P*-Value**	**Standardized coefficient**	**SE**	***P*-Value**
SIE of the percentage of plant protein	0.10	0.01	<0.001	0.04	0.03	0.070	0.10	0.01	<0.001
SIE of the percentage of animal protein	−0.06	0.01	<0.001	−0.02	0.02	0.150	0.01	0.02	0.601
SIE of the percentage of carbohydrate	−0.04	0.01	<0.001	−0.01	0.02	0.674	0.00	0.01	0.824
SIE of the EI^1^	−0.08	0.03	0.002	−0.04	0.02	0.003			
Total indirect effect	−0.09	0.03	0.004	−0.03	0.03	0.346	0.11	0.01	<0.001
Direct effect	0.12	0.01	<0.001	0.01	0.03	0.813	0.13	0.01	<0.001
Total effect	0.03	0.03	0.323	−0.02	0.03	0.534	0.23	0.01	<0.001

*^1^The energy intake without alcohol is not included in the path analysis model for the HiDiet because by construction the HiDiet is made independent of energy intake. All regression coefficients are standardized*.

*SIE, Specific indirect effect; EI, energy intake; AS, adequacy subscore of the PANDiet; SE, standard error*.

*Standardized regression coefficients were estimated with their standard errors by bootstrap resampling using the lavaan package (38). A coefficient of β for a pathway A to B means that a change of 1 standard deviation (SD) in A translates into a change of β SD in B*.

Figure 1 shows that the percentages of energy from plant protein (%PV) and animal protein (%PA) were both significantly associated (at a similar strength) with the AS. A 1 SD increase in the %PV was associated with a 0.19 SD increase in the AS (*P* < 0.001), while a 1 SD increase in the %PA was associated with a 0.22 SD increase in the AS (*P* < 0.001) and a 1 SD increase in the EI (*P* < 0.001) with a 0.76 SD increase in the AS (*P* < 0.001) (Figure 1A). In contrast, a 1 SD increase in the percentage of EI from carbohydrates was associated with a 0.17 SD decrease in the AS (*P* < 0.001) (Figure 1A). Finally, the specific indirect effect (SIE) of the %PV was positive (0.10 SD, *P* < 0.001), whereas the SIE of the other variables was negative: −0.06 SD for the %PA, −0.04 SD for the percentage of carbohydrate, and −0.08 SD for the EI (*P* = 0.002) (Table 2). By combining these SIE, the total indirect effect (TIE) could then be calculated. In this AS model, the TIE was negative, with an increase by 1 SD of the PDI that decreased the AS by 0.09 SD through the TIE (*P* = 0.003) (Table 2). However, the DE was positive, whereby an increase in 1 SD of the PDI increased the AS by 0.12 SD (*P* < 0.001) (Table 2).

A similar model using the SecDiet as the diet quality score (Figure 1B) only showed a significant effect between the EI and the SecDiet, where a 1 SD increase in the EI was associated with a 0.39 SD increase in the SecDiet (*P* < 0.001). The indirect effect mediated by plant protein tended to be significant (0.04 SD, *P* = 0.070) (Table 2). The only significant SIE was the effect through the EI. A 1 SD increase in the PDI was associated with a 0.04 SD decrease in the SecDiet through the EI (*P* = 0.002) (Table 2).

For the third model using the HiDiet as the diet quality score (Figure 1C), the only significant SIE was that mediated by the %PV with a 0.10 SD increase in the HiDiet for each 1 SD increase in PDI (*P* < 0.001) (Table 2). This was due to the significant effect between the %PV and the HiDiet (0.20 SD, *P* < 0.001). In this model, the TIE and the DE were significant and positive (0.11 and 0.13 SD, respectively) (Table 2).

The following models (A2 and S2) estimated the specific effects mediated by each group of plant and animal proteins. In these models, the PDI was associated with changes in protein groups ranging from −0.14 SD for eggs to 0.25 SD for fruits-vegetables-legumes (Figure 2).

Regarding the AS model, a 1 SD increase in whole grains, eggs, fish, and fruits-vegetables-legumes proteins was associated with 0.12, 0.05, 0.18, and 0.09 SD increases in the AS, respectively (Figure 2A). Overall, three SIEs of protein groups were significant. As shown in Table 3, a 1 SD increase in the PDI was associated with a 0.02 SD increase in the AS through the increase in whole grains protein and a 0.02 SD increase through the increase in fruits-vegetables-legumes protein. A 1 SD increase in the PDI was also associated with a 0.01 SD decrease of the AS through the decrease in eggs protein, a 0.05 SD decrease through the increase in the percentage of EI from carbohydrates, and a 0.09 SD decrease through the decrease in EI.

**Table 3:** Variables of the path analyses between the PDI and the two-diet quality score (SecDiet, AS) with whole grains protein (percentage of total energy intake, %E), refined grains protein (%E), dairy protein (%E), eggs protein (%E), beef/sheep meat protein (%E), pork protein (%E), poultry protein (%E), processed meat protein (%E), fish protein (%E), fruits-vegetables-legumes protein (%E), carbohydrates (%E), and energy intake without alcohol as mediators.

**Effects**	**AS model (A2)**			**SecDiet model (S2)**		
	**Standardized coefficient**	**SE**	***P*-Value**	**Standardized coefficient**	**SE**	***P*-Value**
SIE of the percentage of beef/sheep meat protein	0.00	0.01	0.877	0.00	0.01	0.830
SIE of the percentage of processed meat protein	0.00	0.01	0.758	0.00	0.01	0.922
SIE of the percentage of pork protein	0.00	0.01	0.851	0.00	0.01	0.994
SIE of the percentage of poultry protein	0.00	0.01	0.975	0.00	0.01	0.640
SIE of the percentage of dairy protein	0.00	0.01	0.781	0.00	0.01	0.723
SIE of the percentage of eggs protein	−0.01	0.01	0.040	−0.01	0.01	0.014
SIE of the percentage of fish protein	−0.01	0.01	0.113	0.00	0.01	0.513
SIE of the percentage of refined grains protein	0.00	0.01	0.594	0.00	0.01	0.458
SIE of the percentage of whole grains protein	0.02	0.01	<0.001	0.01	0.01	0.130
SIE of the percentage of fruits-vegetables-legumes protein	0.02	0.01	<0.001	0.01	0.01	0.034
SIE of the percentage of carbohydrate	−0.05	0.01	<0.001	0.01	0.01	0.588
SIE of the EI	−0.09	0.03	0.001	−0.04	0.01	0.002
Total indirect effect	−0.11	0.01	<0.001	−0.03	0.03	0.314
Direct effect	0.14	0.01	<0.001	0.01	0.03	0.835
Total effect	0.03	0.03	0.323	−0.02	0.03	0.533

*All regression coefficients are standardized*.

*SIE, Specific indirect effect; EI, energy intake; AS, adequacy subscore of the PANDiet; SE, standard error*.

*Standardized regression coefficients were estimated with their standard errors by bootstrap resampling using the lavaan package (38). A coefficient of β for a pathway A to B means that a change of 1 standard deviation (SD) in A translates into a change of β SD in B*.

Regarding the SecDiet, a 1 SD increase in the PDI was associated with a 0.01 increase in the SecDiet through the increase in fruits-vegetables-legumes protein, and with 0.01 and 0.04 decrease through the decrease in eggs protein and the EI, respectively (Figure 2B).

## Discussion

One significant finding of this study is that the favorable effects of a higher adherence to plant-based diets are dependent on the associated change in plant protein. We found that plant protein mediates significantly the relationship between PDI and two of the three study dimensions, namely, overall positive adequacy (such as coverage of a wide set of nutrient reference values) and long-term health (such as captured with an index of the risk of mortality from chronic diseases). The mediating effect of plant protein between PDI and the nutrient security (such as the risk of deficiency), though non-significant, showed a similar trend.

As explained in the introduction, path analysis does not require a causal relationship and it can be used to account for multicollinearity and identify the importance of some factors in a relationship between two variables (22). It is also important to note that the path analyses' approach is useful to study the different mediation pathways for an association even if the global association is not significant. Indeed, it could help explain how some mediation pathways convey opposite effects that result in a non-significant association between the PDI and the dietary scores.

As expected, our models showed that higher PDI was associated with higher plant protein and lower animal protein intakes. The same result is found in Table 1 with a higher PDI quartile having a higher plant protein intake than the lower quartiles. Thus, higher PDI tended to rebalance plant vs. animal protein intake. A higher PDI was also associated with lower intakes of fat and total energy.

The decreased EI associated with a higher PDI had a negative indirect effect on nutrient diet quality scores. This means that some foods rich in energy and nutrients are consumed to a lower extent in plant-based diets, irrespective of the proportion of energy from macronutrients in the diets. This could be explained by the fact that people with more plant-based diets tend to be more health-conscious, but it may also come from the lower energy density of several plant-based foods. This result shows that deciphering the protein profile from the overall quantitative effect was an important feature of our models. Although this inverse relationship between lower EI with higher PDI is potentially beneficial for health outcomes (46), without precise estimates on physical activity, the specific relationship between PDI and energy balance could not be assessed in this study. Similarly, differences in EIs were not accounted for when scoring long-term mortality and morbidity risk.

Our findings on the importance of plant protein in mediating the benefits of diets higher in plants are in line with other previous studies reporting associations between total plant protein intake and associated nutrients (47, 48). In fact, this study was able to decipher the importance of plant protein within the associated changes in macronutrient and EI, as well as comparatively analyze the contributive mediations. Nutrients conveyed by plant protein intake, known as the protein package, have also been discussed as possibly explaining the benefits of a higher intake of plant protein (21). In this study, we found that plant protein had a favorable impact on both nutrient adequacy and long-term risk, lending credence to the importance of the plant protein package (49–51).

Our study contributes more detailed findings to the plant protein-level type. We found that within plant protein, the proteins from the fruits-vegetables-legumes protein food groups and whole grains were the most salient plant protein mediators of nutrient adequacy and security. Note that only the protein foods as defined previously are considered in the fruits-vegetables-legumes protein food group. We grouped together fruits, vegetables, and legumes to create a protein food category whose contribution to protein intake is low but not negligible (4.6% in the lowest PDI quartile to 6.8% in the highest PDI quartile), so this category should be considered a whole. Higher consumption of these food products in diets with higher PDI was expected since they are part of the items weighted in the score (18), and our results confirm the practical importance of these food sources that are nutrient-dense (52). This is in contrast to refined grains, which are more contributive to protein intake in higher PDI diets, and for which no positive mediation was found. It has been pointed out that refined grains are nutrient-poor and, therefore, impede an improvement in the quality of observed or modeled diets richer in plant protein (53, 54). An increase in nutrient quality with higher PDI should be ascribed in part to fiber. This is mostly available in whole grains and fruit-vegetables-legumes and also mediates part of the decrease in risk of chronic diseases. The protein intake from these groups is that of protein foods; therefore, the mediation found here cannot be ascribed simply to the low density of protein in the fruit-vegetable-legumes group. The nutrient adequacy thus increased with the intake of these food groups. Other authors have analyzed which food categories should be prioritized to fill common micronutrient gaps, and found animal sources only, except dark green leafy vegetables (55). Similarly, previous study has pointed out the importance of animal protein sources in the nutrient intake (11). In our specific population, and analyzing nutritional adequacy and security at the overall diet level, we found that the fruit-vegetable-legumes protein group and the whole grains protein group are important levers in transitioning to a more plant-based diet to secure nutrient adequacy. Beyond nutrient adequacy, relationships between the consumption of these groups and the risk of mortality have been well established (56–58), especially that of cardiovascular diseases (56, 59–61). Altogether, we conclude that the consumption of these food groups, which are contributing to long-term health, is important to prevent a potential reduction of nutrient adequacy in plant-based diets.

While the percentage of animal protein expectedly decreased in diets higher in plants, a mixed relationship was found with the diet quality scores. We found a negative mediation by animal protein on the nutrient adequacy of the diet; thus, the decrease in animal protein limits the improvement in nutrient adequacy. Animal foods, which are rich in protein, are known to make an important contribution to the intake of some vitamins and minerals. This is particularly the case for nutrients conveyed in suboptimal amounts compared to the reference values. Bioavailable iron and zinc, calcium, iodine, and B12 are the most often mentioned (11, 62). In our detailed models, we identified that animal proteins from eggs and fish were salient contributors to the negative mediation by animal protein on the overall positive adequacy subscore.

Results on the nutrient security differed from that on the overall nutrient adequacy since the decrease in animal protein intake in diets higher in plants had no adverse effect on nutrient security. Given that the nutrient security score that we used reflects the changes in the probability of overt deficiency in some nutrients, these findings suggest that animal protein improves nutrient intakes over suboptimal levels. In other words, their relative decrease in a western country, shifting toward a more plant-based diet, would not be expected to lead to nutrient intakes that are notably below the levels of overt deficiency risks. In this regard, it is important to note that we found that lower EI was the most significant contributor to the decrease in nutrient security when diets are more plant-based. This shows that when overt deficiency is concerned, a decrease in energy is more important than the source of protein in the diet. In more detail, we found that animal protein intake from eggs has a more salient beneficial weight on deficiency risk, which may be due to the high contribution of its protein package to iodine, riboflavin, retinol, and B12 intakes (63, 64). It also suggests that this protein source should be given a preference over other animal protein sources when shifting toward a diet with fewer animal products. This is also in agreement with the fact that eggs, in contrast to red meat, are rarely associated with adverse effects on long-term health (65–69).

It should be noted that all results only apply to a Western adult population with a protein intake mainly coming from animal protein and having no risk of protein or amino acid inadequacy, even in the highest PDI quartile (57% of the proteins are animal protein, with a range between 18 and 91% in the total population) (30). These results may be different in a population with a predominantly plant-based diet. However, because they are based on (moderately) plant-based diets that are currently well represented in the population, our results are useful for studying moderate shifts toward these diets that are more likely to occur in the short/medium term in general populations. From a long-term perspective, further studies are needed on diets more plant-based and with much lower animal protein to study the importance of plant/animal protein in nutrient adequacy and long-term health.

As for all structural models, path analyses require evaluating whether data are compatible with pre-specified hypothetical models. However, this methodological approach is not designed to generate alternative structural hypotheses. Moreover, our models had a large number of parameters to estimate with respect to the number of observed variables and were all saturated so that goodness-of-fit or comparison of the present models with more parsimonious ones could not be assessed (Supplemental Table 4).

When building the present model, we had to exclude either lipids or carbohydrates to avoid a set of variables that would be perfectly collinear. However, we tested alternate models (with lipids instead of carbohydrates) and found similar results.

To the best of our knowledge, it is the first study showing that proteins mediate the relationship between the PDI on diet quality scores. It also compares three dimensions of diet quality, providing a complete account of the effects of diets higher in plants. Notably, analysis of the positive or negative mediations by protein food groups also suggests that the quality of a plant-based diet can be further improved by selecting the most efficient changes in terms of protein pattern.

## Conclusion

This study shows the importance of plant protein for the quality of diets that are high in plant content. In particular, it highlights the importance of the nutrient package associated with protein from fruits-vegetables-legumes and whole grains within the plant protein pattern. Lower animal protein intake in more plant-based diets is associated with a decrease in the risk for long-term health but conversely tends to limit the benefit of a higher plant protein intake on the overall nutrient adequacy, although not on nutrient security. It should be noted, however, that eggs protein was found to have a significant positive effect on diet quality. By examining different dimensions of diet quality and deciphering the relationship with a plant-based diet, this study offers a comprehensive view on the importance of protein sources and insights into the best nutritional options when shifting to plant-based diets.

## Data Availability Statement

Publicly available datasets were analyzed in this study. This data can be found here: https://www.data.gouv.fr/fr/datasets/donnees-de-consommations-et-habitudes-alimentaires-de-letude-inca-3/.

## Author Contributions

EP, NL, and FM designed the research. EP conducted the research and drafted the first version of the manuscript. EP, NL, JW, and FM analyzed the data. JW, MS, and J-FH provided methodological support. EP and FM wrote the manuscript and had primary responsibility for the final content. All authors read and approved the final manuscript and provided critical comments on the manuscript.

## Conflict of Interest

The authors declare that the research was conducted in the absence of any commercial or financial relationships that could be construed as a potential conflict of interest.

## Publisher's Note

All claims expressed in this article are solely those of the authors and do not necessarily represent those of their affiliated organizations, or those of the publisher, the editors and the reviewers. Any product that may be evaluated in this article, or claim that may be made by its manufacturer, is not guaranteed or endorsed by the publisher.

## Abbreviations

AS: adequacy score from the PANDiet
CIQUAL: French Center information sur la Qualité des Aliments
DE: direct effect
EpiDiet: evaluate the potential impact of a diet
HiDiet: individual version of the EpiDiet
PANDiet: diet quality index based on the probability of adequate nutrient intake
%PA: percentage of energy from animal protein
PBD: plant-based diets
PDI: plant-based diet index
%PV: percentage of energy from plant protein
SecDiet: index assessing the nutrient Security of Diet
SIE: specific indirect effect
EI: total energy intake
TIE: total indirect effect.
