# Supplementary material for: Plant and Animal Protein Intakes Largely Explain the Nutritional Quality and Health Value of Diets Higher in Plants: A Path Analysis in French Adults
Source: Front Nutr. 2022 Jun 28;9:924526. doi: 10.3389/fnut.2022.924526 (PMC9274246; doi:10.3389/fnut.2022.924526)
Supplement: Supplementary file 1 [file Data_Sheet_1.docx]

Perraud et al.

SUPPLEMENTAL MATERIAL

Supplementary Material

Supplemental Figure 1 Flow chart explaining the sampling of French participants from the third Individual and National Study on Food Consumption Survey (INCA3)


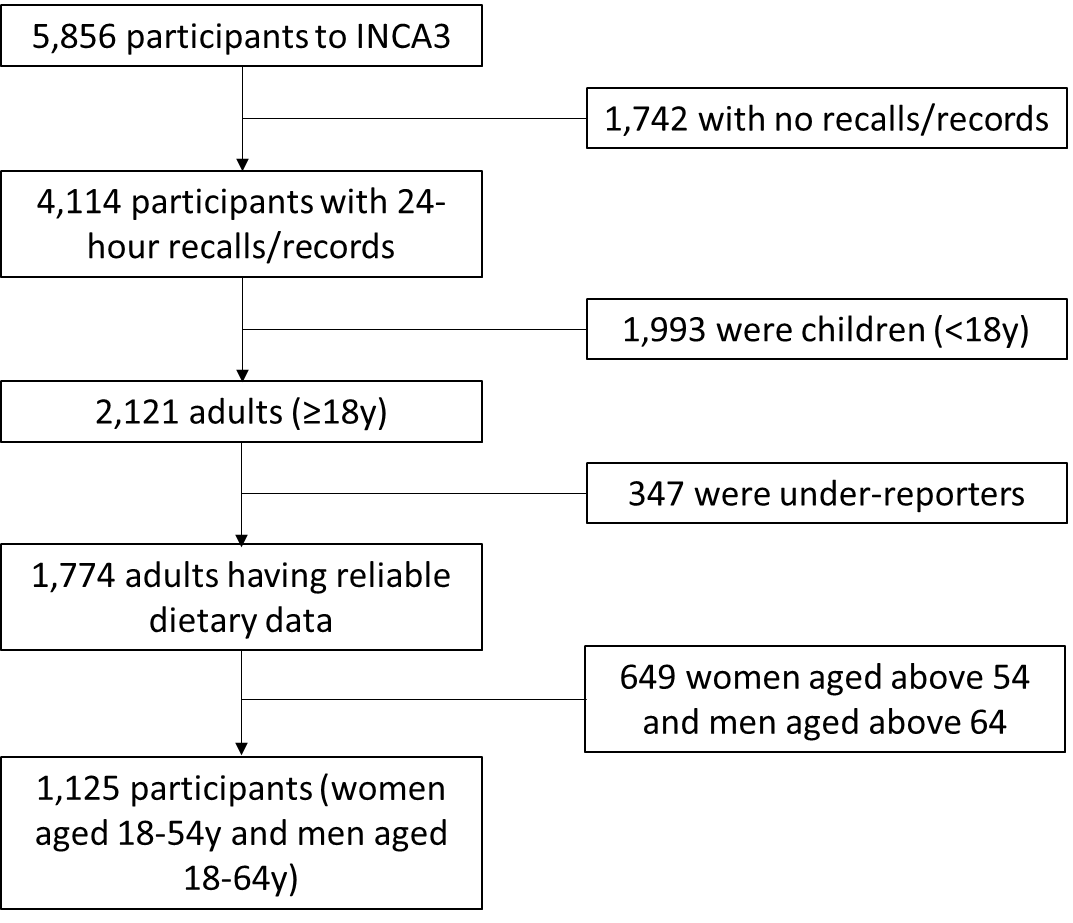


Supplementary Material

Plant-based diet index (PDI)

Mediators i

Diet quality score

α_i_

β_i_

DE

DE: Direct Effect

SIE_i_ = α_i_ + β_i_: Specific Indirect Effect i

TIE = ∑(SIE_i_): Total Indirect Effect

TE = DE + TIE: Total Effect

+ Estimation of the covariances between mediators

- SecDiet
- AS
- HiDiet

Mediators i +1

α_i+1_

β_i+1_

Supplemental Figure 2 Description of path analyses terminology

Supplementary Material

Supplemental table 1 Pearson correlation between the mediators of model A1, S1, and H1

|  | **% plant protein** | **% animal protein** | **% carbohydrates** | **EI** |
| --- | --- | --- | --- | --- |
| **% plant protein** | 1 | -0.47 | 0.48 | 0.02 |
| **% animal protein** | -0.47 | 1 | -0.66 | -0.21 |
| **% carbohydrates** | 0.48 | -0.66 | 1 | 0.15 |
| **EI** | 0.02 | -0.21 | 0.15 | 1 |

%: Percentage of, EI: total energy intake
Models A1, S1, and H1 have the percentage of energy intake as carbohydrates, animal protein, and plant protein as mediators. The energy intake is a mediator only in models A1 and S1.
Model A1 has the AS as diet quality score, model S1 has the SecDiet as quality score, and model H1 has the HiDiet as quality score.

Supplementary Material

Supplemental table 2 Pearson correlation between the mediators of model A2 and S2

|  | **% beef / sheep meat protein** | **% processed meat protein** | **% pork protein** | **% poultry protein** | **% dairy protein** | **% eggs protein** | **% fish protein** | **% refined grains protein** | **% whole grains protein** | **% fruits - vegetables - legumes protein** | **% carbohydrates** | **EI** |
| --- | --- | --- | --- | --- | --- | --- | --- | --- | --- | --- | --- | --- |
| **% beef / sheep meat protein** | 1 | -0.06 | -0.04 | -0.02 | -0.07 | -0.01 | 0.08 | -0.07 | 0.01 | 0.36 | -0.15 | -0.18 |
| **% processed meat protein** | -0.06 | 1 | 0.02 | -0.05 | 0.01 | -0.07 | 0.13 | 0.04 | 0.00 | 0.10 | -0.20 | -0.05 |
| **% pork protein** | -0.04 | 0.02 | 1 | -0.10 | 0.04 | 0.02 | 0.08 | 0.00 | 0.02 | 0.18 | -0.13 | -0.10 |
| **% poultry protein** | -0.02 | -0.05 | -0.10 | 1 | 0.01 | 0.00 | 0.04 | 0.01 | 0.04 | 0.30 | -0.09 | -0.18 |
| **% dairy protein** | -0.07 | 0.01 | 0.04 | 0.01 | 1 | 0.11 | 0.00 | 0.05 | 0.05 | 0.09 | -0.13 | -0.13 |
| **% eggs protein** | -0.01 | -0.07 | 0.02 | 0.00 | 0.11 | 1 | 0.04 | 0.12 | 0.13 | 0.25 | -0.16 | -0.16 |
| **% fish protein** | 0.08 | 0.13 | 0.08 | 0.04 | 0.00 | 0.04 | 1 | -0.25 | 0.08 | -0.05 | 0.12 | -0.09 |
| **% refined grains protein** | -0.07 | 0.04 | 0.00 | 0.01 | 0.05 | 0.12 | -0.25 | 1 | 0.09 | 0.03 | -0.12 | -0.10 |
| **% whole grains protein** | 0.01 | 0.00 | 0.02 | 0.04 | 0.05 | 0.13 | 0.08 | 0.09 | 1 | -0.05 | -0.10 | -0.12 |
| **% fruits - vegetables - legumes protein** | 0.36 | 0.10 | 0.18 | 0.30 | 0.09 | 0.25 | -0.05 | 0.03 | -0.05 | 1 | -0.66 | -0.21 |
| **% carbohydrates** | -0.15 | -0.20 | -0.13 | -0.09 | -0.13 | -0.16 | 0.12 | -0.12 | -0.10 | -0.66 | 1 | 0.15 |
| **EI** | -0.18 | -0.05 | -0.10 | -0.18 | -0.13 | -0.16 | -0.09 | -0.10 | -0.12 | -0.21 | 0.15 | 1 |

%: Percentage of, EI: total energy intake
Models A2 and S2 have the energy intake and the percentage of energy intake as carbohydrates and those from the protein food groups as mediators.
Model A2 has the AS as diet quality score and model S2 has the SecDiet as quality score.

Supplementary Material

Supplemental table 3 Characteristics of the final sample

| Characteristic | | Mean ± SD or n (%) | Theoretical range |
| --- | --- | --- | --- |
| Age (years) |  | 39,9 ± 13,2 |  |
|  | 15-24 | 97 (8%) |  |
|  | 25-34 | 230 (21%) |  |
|  | 35-44 | 316 (28%) |  |
|  | 45-54 | 333 (30%) |  |
|  | 55-64 | 149 (13%) |  |
| Sex |  |  |  |
|  | Men | 564 (50%) |  |
|  | Women | 561 (50%) |  |
| Body mass index (BMI) |  | 24,9 ± 4,6 |  |
|  | BMI < 18.5 | 34 (3%) |  |
|  | 18.5 ≤ BMI < 25 | 610 (54%) |  |
|  | 25 ≤ BMI < 30 | 352 (31%) |  |
|  | 30 ≤ BMI | 129 (11%) |  |
| Education | < college education | 563 (50%) |  |
|  | ≥ one year of college | 562 (50%) |  |
| Region | Île-de-France (Paris Region) | 160 (14%) |  |
|  | North-West | 251 (22%) |  |
|  | North-Est | 282 (25%) |  |
|  | South-Est | 234 (21%) |  |
|  | South-West | 198 (18%) |  |
| Town size | < 2 000 | 287 (26%) |  |
|  | 2 000 - 19 999 | 211 (19%) |  |
|  | 20 000 - 99 999 | 134 (12%) |  |
|  | ≥ 100 000 | 350 (31%) |  |
|  | Paris conurbation | 143 (13%) |  |
| SecDiet |  | 0.958 ± 0.070 | 0 - 1 |
| PANDiet (AS) |  | 68.0 ± 12.0 | 0 - 100 |
| HiDiet |  | 0.006 ± 0.117 | -1 - 1 |
| Plant-based Diet Index |  | 35.6 ± 5.1 | 0 - 90 |

Supplementary Material

Supplemental table 4 Fit indices of the path analyses models between the plant-based diet index and diet quality scores

| **Model** | **SRMR** | **CFI** | **TLI** |
| --- | --- | --- | --- |
| **A1** | 0.000 | 1.0 | 1.0 |
| **S1** | 0.000 | 1.0 | 1.0 |
| **H1** | 0.000 | 1.0 | 1.0 |
| **A2** | 0.066 | 0.83 | 1.0 |
| **S2** | 0.000 | 1.0 | 1.0 |

SRMR: Standardized Root Mean Square Residual, CFI: Comparative Fit Index, TLI: Tucker Lewis Index
Models A1, S1, and H1 have the percentage of energy intake as carbohydrates, animal protein, and plant protein as mediators. The energy intake is a mediator only in models A1 and S1.
Models A2 and S2 have the energy intake and the percentage of energy intake as carbohydrates and those from the protein food groups as mediators.
Models A1 and A2 have the AS as diet quality score, models S1 and S2 have the SecDiet as quality score, and model H1 has the HiDiet as quality score.

Supplementary Material

Supplemental method 1: Calculation of the SecDiet

The SecDiet has been designed to assess the risk of nutrient deficiency due to insufficient intake of nutrients for an adult population. This score computes the probability for each nutrient of having a sufficient intake to avoid nutrient deficiency. This is calculated using the standard normal distribution of nutrient requirements, and taking into account the mean intake y, the day-to-day variability of intake SDy², the number of days of dietary record n, the inter-individual variability SD_r_² and the nutrient reference value r, as follows:

$$F\left( \frac{y-r}{\sqrt{{\mathrm{SD}r}^{2}+ \frac{SDy^{2}}{n}}} \right)$$

F(x) is the function returning the probability that an observation from the standard normal distribution is lower than x. The probability for a nutrient ranges from 0 to 1, where 1 represents a 100 % probability that the usual intake is adequate.

The nutrients included as components in the SecDiet score and the associated threshold values are described in the table below

| Nutrient | Threshold | CV(%) |
| --- | --- | --- |
| Vitamin A | 300 µg RE or 270 µg RE | 15 |
| Thiamin | 0.18 mg/4 .184 MJ | 20 |
| Riboflavin | 1.0 mg | 10 |
| Niacin | 4.35 mg NE/4.184 MJ | 10 |
| Folate | 175 µg | 15 |
| Vitamin B12 | 1 µg | 15 |
| Vitamin C | 10 mg | 10 |
| Iodine | 150 µg | 20 |
| Se | 21 µg or 16 µg | 15 |
| Bioavailable Fe | 1.74 mg | 40 |
| Bioavailable Zn | 1.6 mg or 1.3 mg | 15 |
| Ca | 500 mg | 15 |

An extended description of the method is presented in Salomé et al. (1).

Supplementary Material

Supplemental method 2: Calculation of the adequacy subscore of the PANDiet

The adequacy subscore of the PANDiet accounts for 27 nutrients. DHA and EPA+DHA are weighted by a factor of 1/2 as DHA is present twice. Niacin equivalents were calculated as the sum of dietary niacin and 1/60 dietary tryptophan. Version 3.1 of the PANDiet is based on the dietary reference intake from the 2016 Anses opinion(2) and the overall construction of the score has been described elsewhere(3,4).

| Adequacy subscore | | | |
| --- | --- | --- | --- |
| Nutrient | Reference value (/day) | Variability | Source |
| Protein | 0.66 or 0.8 g/kg bw | 12.5% | (5,6) |
| LA | 3.08% EIEA | 15% | (7) |
| ALA | 0.769% EIEA | 15% | (7) |
| DHA | 0.192 g | 15% | (7) |
| EPA + DHA | 0.385 g | 15% | (7) |
| Fibre | 23 g | 15% | (2) |
| Vitamin A | 570 or 490 µg | 15% | (2) |
| Thiamin | 0.3 mg/1000 kcal | 20% | (8) |
| Riboflavin | 1.3 mg | 10% | (8) |
| Niacin | 5.44 mg NE/1000kcal | 10% | (2) |
| Pantothenic acid | 3.62 or 2.94 mg | 30% | (2) |
| Vitamin B-6 | 1.5 or 1.3 mg | 10% | (9) |
| Folate | 250 µg | 15% | (2) |
| Vitamin B-12 | 3.33 µg | 10% | (2) |
| Vitamin C | 90 mg | 10% | (2) |
| Vitamin D | 10 µg | 25% | (2) |
| Vitamin E | 5.8 or 5.5 mg | 40% | (2) |
| Calcium | 860 (<= 24 y.o) or 750 (>24 y.o.) | 15% or 13% | (2) |
| Copper | 1.0 or 0.8 mg | 15% | (2) |
| Iodine | 107 µg | 20% | (2) |
| Bioavailable iron | See below | | (2) |
| Magnesium | 5 mg/kg bw | 15% | (2) |
|  |  |  |  |
| Manganese | 1.56 or 1.39 mg | 40% | (2) |
| Phosphorus | Calcium (mmol) / 1.65  c.f. phosphorus section in de Gavelle et al. (10) | 7.5% + CV Calcium (mg) | (8) |
| Potassium | 2692 mg | 15% | (8) |
| Selenium | 54 µg | 15% | (2) |
|  |  |  |  |
| Bioavailable zinc | 0.642 + 0.038 b.w. | 10% | (2) |

Iron:

We considered the Anses recommendations that are based on the modeling of obligatory losses but applied to bioavailable iron. For men and non-menstruating women, an EAR of 6 mg/d, RDA of 11 mg/d and CV of 40% defined by Anses were therefore used for iron(2). Assuming iron absorption of 16%, the EAR and RDA correspond respectively to 0.95 mg/day and 1.74 mg/day of bioavailable iron. The deficiency threshold was therefore set at 1.74 mg/d of bioavailable iron, with a CV of 40%. For menstruating women, the distribution of their requirements is not normal and was modeled using a lognormal function by adding basal iron losses (normally distributed) to menstrual losses (exponentially distributed, λ=ln(2)/0.28) with a Monte-Carlo simulation on 1000 individuals in the same way as the calculation of the PANDiet(10). Since the combination of basal and menstrual losses followed a lognormal distribution, it was considered that the logarithm of physiological requirements followed a normal distribution (µ=0.18, σ=0.34). As for iodine, the threshold used overestimated the risk of iron deficiency, so based on the prevalence of anaemia in the French population (2% among men and 5.1% among women(11)) we readjusted it by dividing by a factor of 2.5 for men and non-menstruating women and 2.8 for menstruating women in order to obtain a probability of adequacy of 0.98 for men and 0.95 for women in the French national representative survey INCA3. The corrected DT was set at 0.70 mg/day with a CV of 40% for men and non-menstruating women. For menstruating women, the DT was set at 0.83 mg/day.

Bioavailable iron was estimated using a mathematical model. Nonhaeme iron absorption is dependent on individual concentration of serum ferritin and on dietary factors such as vitamin C, meat, fish and poultry, tea, phytate and calcium intakes. Haeme iron absorption is only dependent on serum ferritin levels. The following equation was used to calculate nonhaeme iron absorption(12):

$$Ln Absorption \left( \% \right)= 6.294 - 0.709 ln \left( \mathrm{SF} \right)+ 0.119 ln \left( C \right)+ 0.006 ln \left( MFP + 0.1 \right)-0.055\ln\left( T+0.1 \right)-0.247\ln\left( P \right)-0.137\ln\left( Ca \right)-0.083 ln(NH)$$

For haeme iron absorption, the following equation was used (13):

$$Log Absorption \left( \% \right)= 1.9897 - 0.3092 \times log (SF)$$

where SF is serum ferritin (mg/L), C is vitamin C (mg), MFP is meat, fish, and poultry (g), T is tea (number of cups), P is phytate (mg), Ca is calcium (mg), and NH is nonhaeme iron (mg).

Because no data were available on the serum ferritin status of individuals, the serum ferritin level was set at 15 mg/L corresponding to the cut-off value for low ferritin stores. However, this hypothesis overestimates the absorption, since iron absorption is maximal at low ferritin stores.

1. Salomé M, Kesse-Guyot E, Fouillet H, Touvier M, Hercberg S, Huneau JF, et al. Development and evaluation of a new dietary index assessing nutrient security by aggregating probabilistic estimates of the risk of nutrient deficiency in two French adult populations. Br J Nutr. 2021;126(8):1225‑36.

2. ANSES. Actualisation des repères du PNNS : élaboration des références nutritionnelles. Maisons-Alfort, France: French Agency for Food, Environmental and Occupational Health Safety (Anses). [Internet]. 2016 [cité 13 mai 2022]. Disponible sur: https://www.anses.fr/fr/system/files/NUT2012SA0103Ra-2.pdf

3. Verger EO, Mariotti F, Holmes BA, Paineau D, Huneau JF. Evaluation of a Diet Quality Index Based on the Probability of Adequate Nutrient Intake (PANDiet) Using National French and US Dietary Surveys. PLOS ONE. 3 août 2012;7(8):e42155.

4. de Gavelle E, Huneau JF, Fouillet H, Mariotti F. The Initial Dietary Pattern Should Be Considered when Changing Protein Food Portion Sizes to Increase Nutrient Adequacy in French Adults. J Nutr. 1 mars 2019;149(3):488‑96.

5. Food and Agriculture Organization of the United Nations, éditeur. Dietary protein quality evaluation in human nutrition: report of an FAO expert consultation, 31 March-2 April, 2011, Auckland, New Zealand. Rome: Food and Agriculture Organization of the United Nations; 2013. 66 p. (FAO food and nutrition paper).

6. ANSES. Anses Opinion on the updating of the PNNS dietary guidelines for women from menopause and men over 65 years of age. [Internet]. 2019 [cité 13 mai 2022]. Disponible sur: https://www.anses.fr/en/system/files/NUT2017SA0143EN.pdf

7. ANSES. Actualisation des apports nutritionnels conseillés pour les acides gras. Maisons-Alfort, France: French Agency for Food, Environmental and Occupational Health Safety (Anses). [Internet]. 2011 [cité 13 mai 2022]. Disponible sur: https://www.anses.fr/fr/system/files/NUT2006sa0359Ra.pdf

8. EFSA. Dietary reference values | EFSA [Internet]. 2021 [cité 13 mai 2022]. Disponible sur: https://www.efsa.europa.eu/en/topics/topic/dietary-reference-values

9. EFSA. Dietary Reference Values for vitamin B6 | EFSA [Internet]. 2016 [cité 13 mai 2022]. Disponible sur: https://www.efsa.europa.eu/fr/efsajournal/pub/4485

10. De Gavelle E, Huneau JF, Mariotti F. Patterns of Protein Food Intake Are Associated with Nutrient Adequacy in the General French Adult Population. Nutrients. févr 2018;10(2):226.

11. SPF. Étude de santé sur l’environnement, la biosurveillance, l’activité physique et la nutrition (Esteban 2014-2016). Volet Nutrition. Chapitre Dosages biologiques : vitamines et minéraux [Internet]. 2019 [cité 13 mai 2022]. Disponible sur: https://www.santepubliquefrance.fr/import/etude-de-sante-sur-l-environnement-la-biosurveillance-l-activite-physique-et-la-nutrition-esteban-2014-2016-.-volet-nutrition.-chapitre-dosages

12. Armah SM, Carriquiry A, Sullivan D, Cook JD, Reddy MB. A Complete Diet-Based Algorithm for Predicting Nonheme Iron Absorption in Adults. J Nutr. 1 juill 2013;143(7):1136‑40.

13. Hallberg L, Hulthén L. Prediction of dietary iron absorption: an algorithm for calculating absorption and bioavailability of dietary iron. Am J Clin Nutr. mai 2000;71(5):1147‑60.
